# A Review of Recent Development of Wearable Triboelectric Nanogenerators Aiming at Human Clothing for Energy Conversion

**DOI:** 10.3390/polym15030508

**Published:** 2023-01-18

**Authors:** Yu Peng, Zheshan Wang, Yunfei Shao, Jingjing Xu, Xiaodong Wang, Jianchen Hu, Ke-Qin Zhang

**Affiliations:** 1 National Engineering Laboratory for Modern Silk, College of Textile and Clothing Engineering, Soochow University, Suzhou 215123, China; 2 College of Advanced Material Engineering, Jiaxing Nanhu University, Jiaxing 314001, China; 3 i-Lab, Suzhou Institute of Nano-Tech and Nano-Bionics, Chinese Academy of Sciences, No. 398 Ruoshui Road, SEID, Suzhou Industrial Park, Suzhou 215123, China; 4 Shanghai Key Laboratory of Special Artificial Microstructure Materials and Technology, School of Physics Science and Engineering, Tongji University, Shanghai 200092, China

**Keywords:** energy conversion, triboelectric nanogenerators, 1D device, fabric structure, woven, knitted

## Abstract

Research in the field of wearable triboelectric generators is increasing, and pioneering research into real applications of this technology is a growing need in both scientific and industry research. In addition to the two key characteristics of wearable triboelectric generators of flexibility and generating friction, features such as softness, breathability, washability, and wear resistance have also attracted a lot of attention from the research community. This paper reviews wearable triboelectric generators that are used in human clothing for energy conversion. The study focuses on analyzing fabric structure and examining the integration method of flexible generators and common fibers/yarns/textiles. Compared to the knitting method, the woven method has fewer restrictions on the flexibility and thickness of the yarn. Remaining challenges and perspectives are also investigated to suggest how to bring fully generated clothing to practical applications in the near future.

## 1. Introduction

Energy issues are attracting more and more attention due to the continuous growth of energy needs both in industry and in the daily life of people. Beyond the traditional technique of producing energy through fossil fuels, new renewable-energy sources such as wind [1], solar [2], acoustic [3], raindrop energy [4], and ocean energy [5] are being explored as alternatives. Different energy-harvesting and conversion strategies are suited to specific applications. For example, humans now rely heavily on portable electronic devices such as cell phones, tablets, and Bluetooth headsets, which need to be charged frequently. The necessity of frequent charging and the climate restrictions of these flexible energy-consuming devices greatly hinder their practicality, sustainability, and broad-range applications, even for wearable electronics [6]. As a result, continuous power supply has become a hot topic in energy-conversion research. It is estimated that if the motion or working time of a human body is 6 h per day, the peak energy generated by human motion could be up to 3.4 W h. This is enough to fully charge a battery with 3.4 V operation voltage and 1000 mA h capacity, close to the capacity of a smartphone battery [7]. Thus, collecting and converting the biomechanical energy from the human body into energy is a potential way to improve the convenience of sourcing power and offers a promising way to break the bottleneck of inconvenience created by the requirement of frequent charging.

The question is how to convert the mechanical energy of human motion into electricity as efficiently as possible. The wearable triboelectric generator is a new type of power generation device that converts kinetic energy into electric power [8,9,10,11]. Several attempts have been carried out to convert human kinetic energy into electrical energy, and the output of some generators has shown the potential for application in electronic devices [12,13,14]. Given the power output and portability of the devices, integrating such devices into clothes, shoes, hats, bags, or the fabrication of wearable generators (as is our focus here) is an attractive research direction for future device design. For wearable generators, not only is power output important, but wearability is also vital. The characteristics of comfort, breathability, skin sensitivity, washability, and moisture permeability of the fabric are the basic factors used to evaluate the standard of wearability for a given material [15,16,17]. Therefore, even though many two-dimensional generators that can be bent but cannot practically be worn on the human body have been designed, it’s hard to define them as wearable devices because they lack fabric features. In addition, some studies have proposed the concept of a generator that can be utilized in clothes, but actual tests of such a generator on the human body as a garment are absent. As for devices that are parts of garments or integrated into garments, the output performance of their use in real-life scenarios is usually much lower than the output generated in a laboratory [18]. Many factors, including the motion strength, motion frequency, materials, and device structure, all influence the real output of the generators. Focusing on the fabric features, this paper summarizes the works of wearable triboelectric nanogenerators that have been truly tested in human clothing, based on the method of fabric manufacture and fabric structures of the devices.

To meet the needs of human activities, smart wearable devices used for human clothing must be light [19], soft [20], washable [21,22,23], breathable [24], foldable [25], tailorable [26], and stretchable [27] so that they can be directly manufactured or integrated into cloth. Many attempts at manufacturing stretchable fiber-based/fabric-based generators that are able to be attached or jointed to cloth as wearable prototypes have been carried out. At present, due to the defects in the material and/or haptic experience [28], reports on such devices truly being used in human clothing are still limited, and some of the reported textile-based/-shaped devices were far from ideal in collecting human-motion energy [29].

In this review, we summarize the braided structure and performance of triboelectric nanogenerators (TENGs) over the last decade that were integrated into human clothing to collect human-motion energy in real-world circumstances and deeply analyze them from the perspective of the textile structure. Different fabric structure makes the fabrics suitable for different clothing applications. Woven fabric that is tight, windproof, durable, good for draping, and wrinkle resistant is usually used in shirts, jackets, suits, trousers, etc. [30,31]. Knitted fabric that is relatively loose, soft, and elastic; has good breathability [32]; is easily dispersed; and has an edge that is easy to roll is usually used to make underwear, socks, T-shirts, sweaters, leggings, and so on [33,34]. Textile-based generators or woven/knitted structural generators are ideal for wearable devices intended for practical applications in garments. In addition, different properties of yarns, such as the pliability, thickness, length, durability, etc., are suitable for different fabric structures. In light of this and the findings of our research, designing corresponding textile energy devices by using available materials or designing new materials can hopefully be accelerated.

## 2. Smart Wearable Generators Integrated into Garments

Thus far, integrating the devices into garments is the most popular method for fabricating wearable generators for energy capture and conversion. Since the electric materials are usually rigid and are difficult to fabricate into cloth directly, the most common strategy is modifying them into flexible fibers or yarns before manufacturing textile devices. In recent years, many research experiments have focused only on the flexibility of the devices, which is the basic requirement for wearable devices, whereas occasional works have paid attention to comfort assessment, which is strongly linked to the composition of the fabric or textile and is essential to the real use of such wearable generators in context. Many aspects contribute to the haptic experience of textiles, such as wettability, stiffness, smoothness, and so on, and the fabric structure is one of the most influential factors in the comfort assessment [28]. 

**Figure 1:**
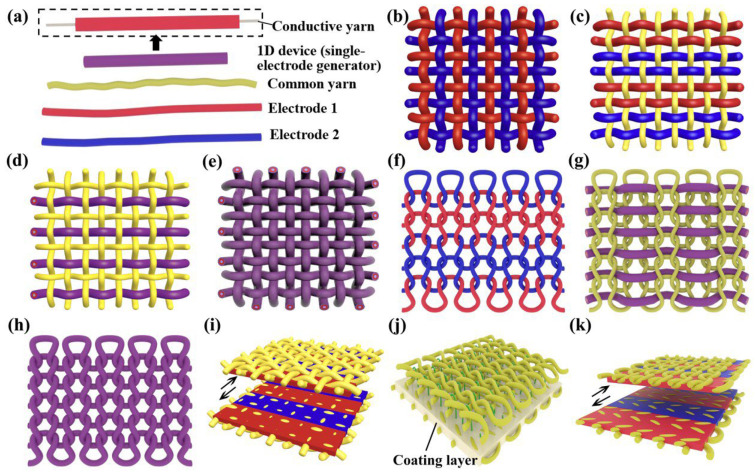
Schematic of the textile-based generators used in human clothing. (**a**) The basic elements of a textile-based generator. (**b**) Woven-structure generator based on two kinds of 1D electrodes. (**c**) Woven-structure generator based on two kinds of 1D electrodes and common yarns. (**d**) Woven-structure generator based on two kinds of 1D devices and common yarns. (**e**) Woven-structure generator based on 1D devices. (**f**) Knitted-structure generator based on two kinds of 1D electrodes. (**g**) 1D device sewn into common knitted fabric. (**h**) Knitted-structure generator based on 1D devices. (**i**) Woven-structure generator based on fabric coated with functional layers. (**j**) 3D spacer fabric-structure generator and (**k**) knitted-structure generator based on fabric coated with functional layers.

The TENG is usually based on two friction materials of opposite polarity, which have opposite charges when subjected to an external force to form an electrical potential. By connecting these two materials with conductive wires, the electrical energy generated by the charge movement is obtained. Some TENGs work with human skin or clothing, acting as one of the friction electrodes, so the TENG is designed to be composed of a single electrode. The fundamental working modes of TENGs can be divided into four categories: vertical contact-separation mode, lateral sliding mode, single-electrode mode, and free-standing mode [35,36,37,38]. Inspired by these, researchers have invented many kinds of fabric-based devices [39,40,41,42,43,44,45,46]. Some of these devices are based on woven structures [47,48,49,50,51] and others on knitted structures [52,53]. We summarize the fabric-based devices into these classifications, as shown in Figure 1: woven-structure TENG based on two types of one-dimensional (1D) electrodes (Figure 1b,c) and 1D devices (Figure 1d,e), knitted-structure TENG based on two types of one-dimensional (1D) electrodes (Figure 1f) and 1D devices (Figure 1g,h), and TENG based on coated fabric (Figure 1i–k).

The yarns in woven fabrics are interwoven and extend horizontally and vertically, so the woven fabric is highly compact and has good wind resistance. The yarns in knitted fabrics exist in the form of nested loops. Each loop can be stretched and extended in all directions, so the knitted fabric has good elasticity and breathability. The differences in fabric properties due to different structures are enormous. Here, we propose a simplified classification of TENG, which contains only two categories. In this paper, we analyze in detail the design and performance of TENGs that have truly been used as part of garments to harvest human kinetic energy over the last decade and discuss the challenges faced by TENGs.

### 2.1. TENGs Based on Woven Textiles

Woven fabric is a textile formed by weaving. Most woven fabrics were produced on a loom and are made of many threads woven on a warp and a weft. TENGs with a structure woven from different weaving units will be discussed in this section.

#### 2.1.1. Woven-Structure Generators Based on Fibers/Yarns

**Figure 2:**
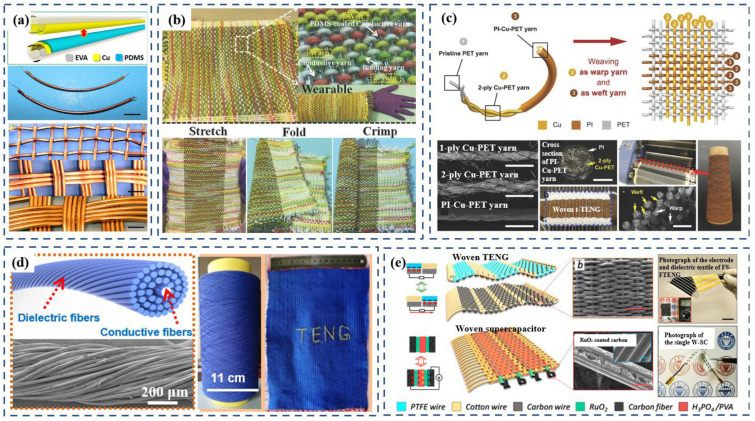
(**a**) TENG fabricated from a Cu-coated ethylene vinyl acetate (EVA) electrode and a polydimethylsiloxane (PDMS) covered Cu-coated EVA tubing electrode. Reproduced under the terms of the CC-BY Creative Commons Attribution 4.0 International License (https://creativecommons.org/licenses/by/4.0) (accessed on 28 September 2022) [54]. Copyright 2016, American Association for the Advancement of Science. (**b**) Digital photographs of a large-area wearable textile TENG (top view). Bottom views are photographs of the TENG under various mechanical deformations, including stretching, folding, and crimping. Reproduced with permission [55]. Copyright 2017, John Wiley and Sons. (**c**) TENG fabricated from Cu-coated PET warp yarns and 2-ply PI-coated Cu-PET weft yarns. Reproduced with permission [56]. Copyright 2016, John Wiley and Sons. (**d**) The core–shell yarn manufactured by 200 elastic spandex fibers tightly twined around two parallel stainless-steel fibers and digital photographs of the TENG. Reproduced with permission [57]. Copyright 2017, American Chemical Society. (**e**) Schematic illustration of the free-standing-mode fabric TENG. Reproduced with permission [58]. Copyright 2018, Elsevier.

The yarns in woven fabric are nearly immobile, leading to an almost unextendible fabric sheet with limited deformations in the yarn structure. Thus, normal woven fabric does not need a great deal of flexibility or bending in the warp and weft threads. Therefore, a rigid, inflexible, but conductive metal electrode can be woven into fabric-based devices as warp or weft, even when they are not that satisfactory for weaving. Many devices have modified the electrode instead of using metal wires directly to increase softness and comfort. In 2016, Wen et al. [54] fabricated a TENG by using a Cu-coated ethylene vinyl acetate (EVA) electrode and a polydimethylsiloxane (PDMS)-covered Cu-coated EVA tubing electrode as warp and weft, respectively. The Cu-coated EVA tubing in this work acts not only as the electrode for the TENG but also as the holder for fabricating a fiber-shaped dye-sensitized solar cell. Therefore, it can also be fabricated into a textile-based supercapacitor to store the electric energy collected by the TENG after rectification. Although the single EVA tubing was flexible, after copper deposition and PDMS coating, the diameter of the single TENG unit was about 3 mm (Figure 2a). The metal yarn-based fabric structure made of such thick yarns makes the device inflexible and bulky. As a result, the device can only be integrated in limited positions on garments, such as by attaching it onto a T-shirt.

Dong et al. [55] overcame the inflexibility of thick yarn-based fabric. In their study, a 3-ply-twisted stainless steel/polyester fiber-blended yarn was used as the warp thread and a PDMS-coated energy-harvesting yarn was used as the weft thread. Although the diameter of the warp and weft yarns was even larger than that of the previously mentioned electrode, the flexibility of the textile was maintained, allowing it to be stretched, folded, or crimped (Figure 2b). This effect relies on two advanced improvements in the materials and structure design. One is that the warp and weft yarns were all extremely flexible and could be stretched, twisted, bent, and knotted. The other improvement is that they interwove Z-directional cotton yarns with the weft yarn along the warp direction to make the fabric soft and skin-friendly. This compound structure can be categorized as the fourth structure in Figure 1d. The cotton yarns not only increase flexibility, but also play a role in absorbing the sweat of human skin or moisture in the environment to balance the humidity between the skin and the outer environment. In tests conducted with the fabric worn on the forearm, the average voltage amplitude reached up to 125 V.

Similarly, Zhao et al. [56] also used twisted yarn to fabricate a functional textile. They designed a TENG that was fabricated by directly weaving together Cu-coated polyethylene terephthalate (PET) warp yarns (300 μm in diameter) and 2-ply polyimide (PI)-coated Cu-PET weft yarns (350 μm in diameter) (Figure 2c) on a weaving loom. They stitched the prepared TENG into a white, nonelastic cotton chest strap and used analogue-to-digital conversion and a filter for monitoring the respiration of the tester. A real-time respiratory pattern could be recorded with four different breathing states, including deep, shallow, rapid, and slow using the prepared textile triboelectric nanogenerators (t-TENGs).

Yu et al. [57] further improved the method of twisting the blended yarns by adopting the stainless-steel fibers as the core and twisting the dielectric fibers to form the sheath, creating core–shell yarns through a commercial machine. The manufactured TENG was obtained by weaving the yarn composed using this method. To fabricate core–shell yarn, 200 elastic spandex fibers were tightly twined around two parallel stainless-steel fibers (Figure 2d). The high ratio of elastic dielectric fibers can reduce the rigidity of the yarn and improve its softness. The resulting TENG textiles are skin-friendly and flexible, and their fabrication processes is compatible with industrial manufacturing technology for large-scale textile production. In addition to the woven-structure textile, they also made a knitted-structure textile whose loops were very loose to collect the kinetic friction. By using this knitted TENG textile, they were able to realize more complicated and fashionable garment designs. A greater number of positions on the human body could be used for attaching the TENG textiles onto clothes. In their test, two pieces of TENG textiles woven from spandex-fiber-based core–shell yarns were sewn under the arm of a sweater and under the thenar of a sock. The output performance of the TENG textile under the thenar was significantly higher than that of the TENG textile under the arm. It achieved outputs as high as ~125 V open-circuit voltage and ~4 mA m^−2^ short-circuit current density when running. 

Unlike the methods discussed above, in which metal coatings or metal wires were used, Chen et al. [58] used commercial polytetrafluoroethylene (PTFE), carbon, and cotton wires to fabricate a TENG, and the process was carried out on a traditional shuttle-flying weaving loom. In this work, they designed the TENG with two different work modes: a vertical contact-separation mode and a lateral sliding mode. For the contact-separation-mode TENG, the electrode textile was prepared by using nonconductive cotton threads as the warp and conductive carbon wires as the weft. A dielectric textile was woven from the carbon warp threads and PTFE weft wires. By assembling a woven supercapacitor into the TENG with a full-wave rectifier, a device was made for harvesting arm-swaying energy that could continually charge an electric watch as a self-powered system (Figure 2e). The long-term stability of the self-powered device was tested in this study, and after 15,000 cycles, ~80% of the original output voltage remained. The mosaic-pattern knitted textile allowed for the flexibility and aesthetic appearance of the fabric device. Additionally, though the underarm is one of the few places on the human body where both contact-separation and sliding modes of generating electricity can be applied, it is also the place where the body is most likely to produce sweat. The use of cotton greatly inhibits the performance of the TENG by exposing it to high humidity and liquid contact [59,60,61]. If the cotton becomes conductive after absorbing moisture when the wearer is in motion or in a humid environment, no power energy will be generated. Therefore, a waterproofing treatment should be considered to improve the device’s performance. Gong et al. [62] invented an amphibious triboelectric textile, which was woven from super stretchable and flexible triboelectric yarns consisting of intrinsically elastic silicone rubber tubes and built-in helical-structure stainless-steel yarns. In this study the single-electrode triboelectric yarn was capable of lighting up a liquid-crystal display device underwater. The triboelectric textile woven from the yarns can be worn on the tester’s elbow and harvest biomechanical energy from bending the elbow. The elbow-bending test confirmed the flexibility and durability of the yarns. Additionally, the ultralong yarns makes it possible for the device to be fabricated on a knitting machine.

The weaving method requires the lowest performance of functional yarns, because even short, coarse, or poorly flexible yarns can be woven into fabrics by the weaving method. As long as the functional yarns are used as the weft yarns and the flexible yarns are used as the warp, a woven fabric can be made. Modifying the fibers/yarns endowed the material with a special function while simultaneously enhancing the stability and life cycle of the fabric device. However, because the modified electronic fibers/yarns were not as soft as traditional yarns, the textile devices became bulky and inflexible. Further effort is needed to fabricate soft, flexible, and functional fibers/yarns for the construction of generator devices. 

#### 2.1.2. Woven-Structure Generators Based on Textile Strips

Beyond the fiber-based/yarn-based woven-structure generators, some studies used textile strips as warp and weft to fabricate textile-based generators. The use of woven fabric strips as units to create new fabrics is a method of fabric production that is unique to weaving. Woven fabric strips usually are soft, have a flat surface, and can be produced in large quantities. The length and width are controllable and can be easily remanufactured into garment fabrics. Using woven fabric strips as the basic unit and modifying them to obtain the desired properties and making functional two-dimensional fabrics from the treated strips is a relatively simple and efficient method. The resulting fabric is as soft and flexible as normal clothes and can easily be integrated into garments. The same method cannot be applied to knitted fabrics because their properties of common dispersion, hemming [34], etc. prevent them from being cut into strips. It is also possible to manufacture knitted strips specifically, but this method is tedious and the resulting individual strips have uneven surfaces and tend to curl, making it difficult to manufacture garments. 

**Figure 3:**
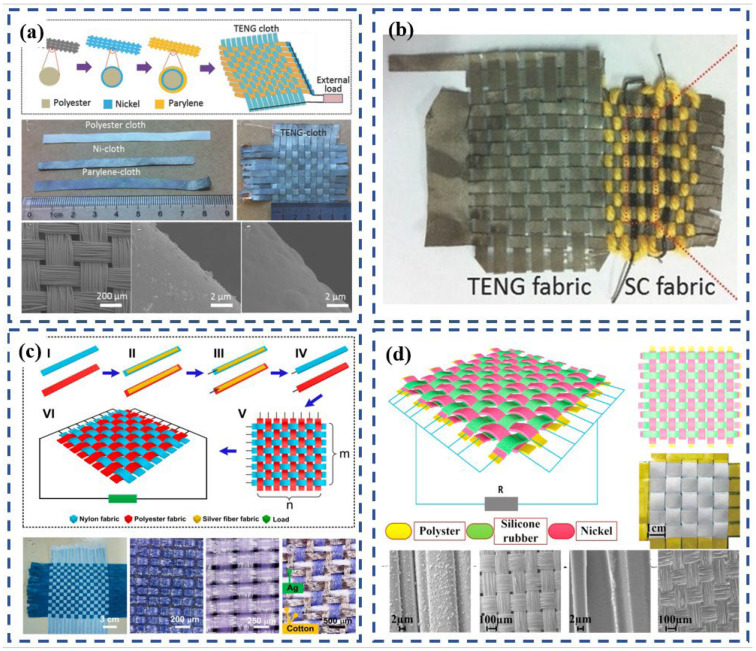
(**a**) TENG formed by using 10 Ni-coated polyester strips as longitude lines and 10 parylene-Ni-coated strips as latitude lines. Reproduced with permission [7]. Copyright 2015, John Wiley and Sons. (**b**) Self-power system that used a fabric-structure TENG and textile-structure supercapacitor. Reproduced with permission [63]. Copyright 2015, John Wiley and Sons. (**c**) TENG made of a commercial nylon-fabric strip, polyester-fabric strip, and homemade conductive silver-fabric strip. Reproduced with permission [64]. Copyright 2014, American Chemical Society. (**d**) TENG manufactured using Ni-coated polyester conductive-textile strips and silicone rubber-Ni-coated polyester strips by the traditional “plain grain” method. Reproduced with permission [65]. Copyright 2017, Elsevier.

In 2015, Pu et al. [7] fabricated a woven TENG by using 10 Ni-coated polyester strips as longitude lines and 10 parylene-Ni-coated strips as latitude lines (Figure 3a). The TENG cloth was worn in different positions on the human body—namely, under the foot, under the arm, and at the elbow joint. When doing activities, the TENG cloth worn under the foot and arm generated enough power to light up 37 and 17 LEDs, respectively. However, this device has some limitations. First, when using this device, it was difficult to integrate the storage of a lithium-ion battery (LIB) into clothing. The safety and resource consumption of the device were also problematic. In 2016 [63], Pu et. al. proposed a new self-powered system that used the same materials and same fabric structure as their previous TENG, but a textile-structure supercapacitor was used instead of the LIB to store the generated power. The supercapacitor shared the same warp system as the TENG, forming a seamless fabric so that the TENG and the supercapacitor became a single unit without seams or imperfections (Figure 3b).

Zhou et al. [64] and Tian et al. [65] focused on the different power outputs generated by different human motions. In Zhou’s work, the source materials for the TENG were a commercial nylon-fabric strip, a polyester-fabric strip, and a homemade conductive silver-fabric strip. The silver strip was pasted in the center of two nylon strips or two polyester strips. The resulting nylon–silver–nylon strips and polyester–silver–polyester strips were used as warp threads and weft threads, respectively, that were woven into a fabric (Figure 3c). The homemade conductive silver-fabric strips made of silver fibers and cotton fibers were shown to greatly improve the durability and life of the device. To demonstrate its potential applications, the textile-structure TENG was integrated into shoes, coats, and trousers to harvest different kinds of mechanical energy from human motions. The currents generated from footsteps, the shaking of clothes, bending leg joints, and bending arm joints were 0.3 μA, 0.75 μA, 0.9 μA, and 0.75 μA, respectively (the effective area of the TENG was 20 cm^2^). These test results further confirmed the TENG’s practical application potential as a wearable device. Tian et al. [65] fabricated a TENG by using Ni-coated polyester conductive textile strips and silicone rubber-Ni-coated polyester strips using a traditional “plain weave” method (Figure 3d). The currents of the TENG fixed under the arm, at the elbow joint, under the foot, and at the knee joint reached 30 μA, 4 μA, 40 μA, and 15 μA, respectively. The thickness of the strip was 750 μm, which caused the device to be bulky and inflexible. Additionally, the device’s thick coating layer eventually fell off following subsequent use of the device, reducing its output and life.

The strip-based woven-structure TENG has a performance similar to that of the fiber-based/yarn-based woven-structure TENG. In addition, it is flexible and durable, making it possible to integrate such fabric devices into human clothing. However, this structure requires two weaving processes, which makes the device-fabrication process more complex and time-consuming than other structural TENGs. For strip-based woven textile devices, the best and only use is to integrate them into clothing as part of the garment for wearable applications. Therefore, it is important to find more ways to make fabric devices that have a simple fabrication process and can be manufactured on a large scale.

#### 2.1.3. Generators Based on Woven Coated Fabric

**Figure 4:**
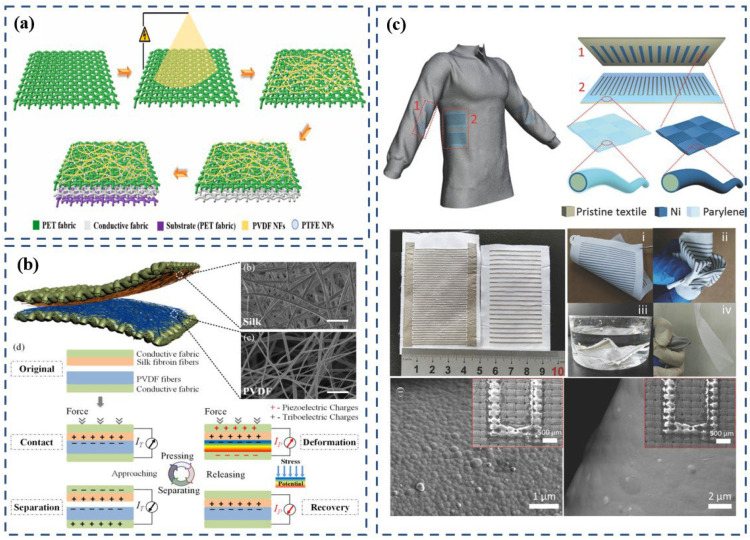
(**a**) Schematic illustration of modified fabrication process of power-generating fabrics. Reproduced with permission [26]. Copyright 2019, Elsevier. (**b**) Schematic diagram of an all-fiber hybrid triboelectric nanogenerator, which consists of two electrodes (conductive fabric) and electrospun silk nanofibers and PVDF nanofibers serving as a triboelectric pair. Schematic view of the operating principle of the hybrid nanogenerator. Reproduced with permission [66]. Copyright 2018, Elsevier. (**c**) Schematic of a power-textile with a pair of TENG fabrics consisting of a slider fabric (1) in the sleeve and a stator fabric (2) underneath the arm. Reproduced with permission [67]. Copyright 2016, John Wiley and Sons.

In 2019, Qiu et al. [26] utilized the electrospinning and electrospray methods to obtain PET fabric coated with PTFE nanoparticles and PVDF nanofibers (Figure 4a). The resulting fabric was then firmly attached to a conductive fabric with double-sided tape. By integrating the power-generating fabric with a daily-wear garment, they found that mechanical energy could easily be harvested from human movement. After biomechanical excitation, this all-fabric textile produced a power density of 80 mW/m^2^ at a load of 50 MΩ. The flexibility, light weight, air permeability, and durability of the power-generating fabric was tested, and the results showed that the power-generating fabric could be applied to clothing. A series of common fabrics such as nylon, silk, cotton, T/C (terylene and cotton mixed to a specific ratio), and PP (polypropylene), along with PET, were chosen as raw materials and served as triboelectric layers to demonstrate the applicability of diverse fabrics in power-generating fabric construction. The results demonstrated that this coating strategy is universally applicable to most commonly used fabrics, and the surface-coating technique successfully modified the composite fabrics. It is worth noting that the power-generating fabrics maintained their basic function even after being cut into pieces. What is more, research found that the device can work well even when tailored, halved, or spliced. This characteristic has exciting potential, opening up the prospect of designing tailor-made, power-generating clothes in different styles and sizes.

From this point of view, electrospinning nanofibers into fabrics is an ideal method for manufacturing wearable devices. Whereas a traditional coat layer reduces the breathability of the fabric [24], the electrospinning coating has the advantage of both air permeability and flexibility, and furthermore allows the surface of the fabric to be modified so it can achieve the desired function [68,69,70,71,72].

Guo et al. [66] also used electrospinning coatings in the manufacture of TENG. They designed a hybrid triboelectric and piezoelectric generator to maximize the collection of human kinetic energy. The hybrid generator consisted of two fabric electrodes: conductive fabric covering silk electrospinning nanofibers and conductive fabric covering PVDF electrospinning nanofibers (Figure 4b). The study demonstrated the applicability of the hybrid generator to monitor human activity and personal medical care, and the cycle stability of the device was also verified. Compared to cold, rigid medical monitors, soft, fabric-based detectors can provide a more comfortable experience for patients.

In addition to the electrospinning method, electroless deposition (ELD) can also be used to fabricate functional coatings for TENG. In fabricating TENG, Pu et al. [67] proposed a laser-scribing mask and the ELD nickel-plating method for synthesizing conductive circuits/patterns on fabrics. They fabricated TENG fabrics with interlaced grating structures (Figure 4c) and integrated them with SC to realize a fabric-based energy-harvesting system. The thin coating of Ni does not significantly increase the weight of the fabric, maintaining the light weight and softness of the pristine textile. As shown in Figure 1d, TENG fabric can be easily bent, wrapped, and immersed in water without damage, which shows that it has good flexibility and that household clothes can be used and washed. In addition, the TENG fabric has been proven to have good air permeability and shape retention. 

The thickness of the coating affects the breathability of the fabric, and the adhesion condition of the coating to the fabric substrate determines the washability of the fabric. Attention needs to be paid to the breathability of the fabric and the adhesion of the coating when designing the wearable TENG. The coating method of the grating structure retains the breathability of original fabric. The use of nanoscale particles and fibers as coatings enhances the adhesion between the coating and the fabric substrate. All these designs are well considered for the performance of the generator based on woven coated fabric. In addition, the lifetime of the coating has to be considered, because once the generator is made/integrated into a garment, the fabric will be affected by high-frequency external forces during wearing or washing.

### 2.2. Generators Based on Knitted Textiles

Knitted fabric created by interlacing yarn in a series of connected loops using straight eyeless needles or by machine has high flexibility and breathability [73,74]. The knitting loops are arranged by suspension in a horizontal (course) or vertical (wale) direction. These meandering and suspended loops can be easily stretched in different directions, meaning they have more elasticity than other types of textiles [75,76]. However, harsh deformations during the fabrication of a knitted fabric can possibly damage the fiber/yarn. During the process of manufacturing knitted fabrics, each loop has to withstand manipulation by mechanical external forces, so the yarn must be durable.

#### 2.2.1. Knitted-Structure Generators Based on Fibers/Yarns

**Figure 5:**
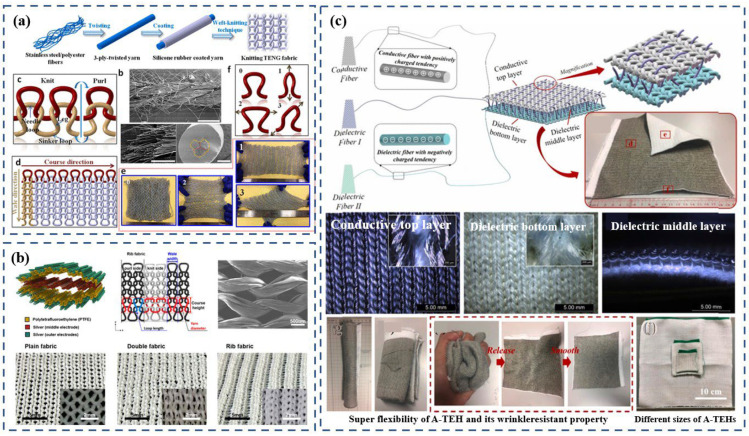
(**a**) Knitted-structure generator based on 1D devices. Reproduced with permission [77]. Copyright 2017, American Chemical Society. (**b**) Knitted-structure generator based on two kinds of 1D electrodes. Reproduced with permission [78]. Copyright 2017, American Chemical Society. (**c**) Schematic illustration of the realization of the energy-harvesting mode as 3D full-fabric structural integrity using all fiber materials by a computerized knitting programming and loop structures. Reproduced with permission [41]. Copyright 2019, Elsevier.

Dong et al. [77] developed a knitted-fabric TENG by using a single energy-harvesting yarn that is fabricated by coating silicone rubber over the surface of three-ply twisted stainless-steel/polyester-fiber blended yarn (Figure 5a). By taking advantage of the weft-knitting technique, the resulting fabric TENG possessed high elasticity, flexibility, and stretchability so it could be elongated, widened, or distorted by external or internal forces in any direction. As a prototype, the knitted power-generating fabric could be worn on the body directly, for example as an insole inside a shoe or as a bracelet worn on the wrist. After 50,000 cycles of repeated contact–separation motion at a contact frequency of 4 Hz, the open-circuit voltage and short-circuit current showed no obvious degradation. The electrical outputs of the knitted TENG fabric experienced no decrease after multiple washing cycles. The study demonstrated that the electrical output performance was positively correlated with yarn diameter. However, the problem is that given the current industry trend of pursuing lightness, thinness, and transparency, a fabric TENG made from yarns that are large in diameter results in thick and bulky clothing. The compatibility of the thick fabric with common fabrics, which tend to be thin, is also an issue. Therefore, the application of wearable devices in real-life situations should be further explored to create a balance between diminished thickness and high output.

Using a different approach than the single-electrode work mode discussed above, Kwak et al. [78] invented a knitted-fabric TENG that harvests motion energy using a contact-separation mode. The TENG was made from five knitted-fabric layers (Figure 5b), which consisted of knitted PTFE fabrics for the top and bottom triboelectric layers and knitted Ag fabrics for the electrode in the middle and on the back of the top and bottom triboelectric layers. They investigated plain-, double-, and rib-fabric structures (Figure 5b) and analyzed their potential for textile-based energy harvesting. Compared with the fabrics’ original states, increasing the amount of surface contact during the stretching is crucial. Although it is possible to extend the plain fabric in both the wale and course directions, its capacity to extend is lower than that of the double and rib structures because the contact between each loop is maintained even during stretching. Double-knitted fabric and rib-knitted fabric showed significantly superior stretchability and output. The latter showed significantly improved stretchability, which dramatically enhanced the triboelectric power-generation’s performance due to the increased contact surface. The durability of the rib-knitted TENG was also superb; the voltage was maintained even after 1800 stretching cycles under 30% strain. After integrating the TENG into a sportscoat and running, the TENG was able to light 15 green LEDs.

Gong et al. [41] adopted a new kind of all-textile energy harvester with three sets of fibers by using computer programming. In this device, the conductive silver-plated nylon fibers with a positively charged tendency were knitted as the top layer, the dielectric polyacrylonitrile (PAN) fibers with a negatively charged tendency formed the bottom layer, and the dielectric PAN fibers without post-treatment were used to directly knit the top layer and bottom layer together (Figure 5c). This compound-structure fabric overcomes the shortcomings of traditional knitted fabric, which is prone to dislodgement and edge curl, and it can be tailored into any desired shape. In their research, a shoe-insole-shaped harvester was tailored to collect human kinetic energy. When the fabric becomes dirty, leading to performance degradation, the electric power generation can be recovered again after simply rinsing the fabric with tap water like common clothes and air-drying it. The textile energy harvester’s wearable performance, including its comfort, breathability, washability, and unique advantage of tailorability, lends it to versatile product design.

The capacity of the knitted loops to stretch and deform greatly improves the elasticity of the fabric device and makes up for the inflexibility of the functional fibers/yarns. Moreover, the modification of the fibers/yarns does not affect the air permeability of the knitted fabric. Compared with a woven device, a knitted device can be softer and more compatible with the design requirements of garments.

#### 2.2.2. Generators Based on Knitted, Coated Fabric

The high elasticity and porosity of knitted fabrics contribute to the breathability and softness of the garment. If a coating is applied to the top of a knitted fabric, when the knitted fabric is being stretched, the breathability of the fabric may be reduced and the adhesion of the coating may be compromised. Much work has been carried out to investigate ways to enhance knitted/coated-based generators’ garment properties, such as breathability, stretchability, and washability.

**Figure 6:**
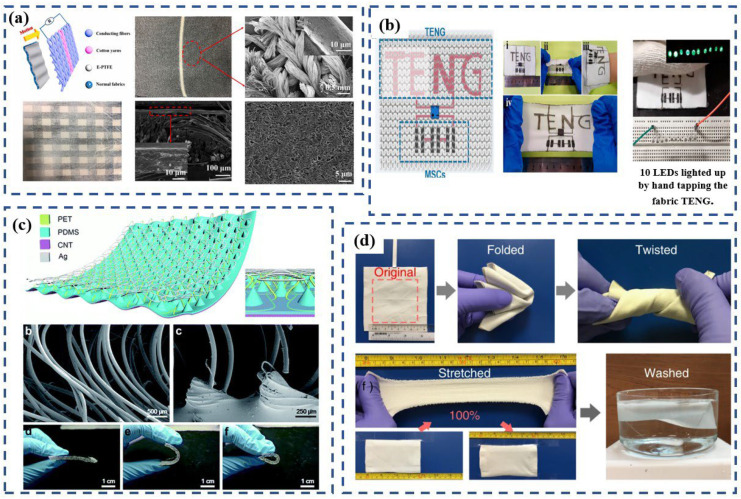
(**a**) The free-standing-mode TENG textile, which is made up of a laminated composite fabric consisting of an expanded polytetrafluoroethylene (E-TFE) film and a common fabric. Reproduced with permission [6]. Copyright 2017, Elsevier. (**b**) Stretchable coplanar self-charging power knitted textiles with a triboelectric nanogenerator (TENG) and microsupercapacitors (MSC). Reproduced with permission [75]. Copyright 2017, American Chemical Society. (**c**) TENG made of 3D-space fabric on the market as the base material by coating PDMS, conductive silver paste, and CNT. Reproduced with permission [79]. Copyright 2017, Royal Society of Chemistry. (**d**) Photographs demonstrating that the textile triboelectric nanogenerator (textile-TENG) possesses excellent endurance for successively experiencing deformations of folding, twisting, and stretching, as well as severe washing. Reproduced under the terms of the CC-BY Creative Commons Attribution 4.0 International License (https://creativecommons.org/licenses/by/4.0) (accessed on 21 September 2022). [80].

Huang et al. [6] studied the influence of different knitted structures on the output performance of TENGs. The TENG they used was fabricated by using a heat-welding adhesive net to coat the expanded PTFE membrane on top of knitted cloth via a thermal-calendaring process (Figure 6a). They designed five types of knitted cloth with different stitch densities on each side. PTFE was coated on to all 10 sides of the five knitted cloths. The result showed that the TENG textiles with higher stitch densities showed a voltage of 900 V and current of 19 μA, which are approximately twice as high as those of alternate sides with lower stitch density, and higher than other types of TENG textiles with lower stich density. Clearly, the superiority of these TENG textiles comes from the larger contact area. The TENG fabric developed during this experiment could be folded like normal clothes. Its good durability was demonstrated from the voltage–current curves of the TENG-textile electrode under deformed states. It retained good conductivity after being twisted or stretched for 500 cycles. There were no obvious changes to its voltage–current curves after it was washed in up to 10 cycles, which shows good washability.

Cong et al. [75] developed a stretchable knitted TENG using the resist dyeing-analogous method. In this method, Ni-coated textile with two in-plane electrodes and an elastomeric PDMS thin layer is coated onto one of the Ni-coated textiles. The TENG generates power when a polyester textile does repeated touching–separating motions. Using the same method, they also invented a microsupercapacitor that was fabricated into the same textile (Figure 6b) with the TENG. The impact of different strains and humidities on the output was studied. After being stretched to 100% strain along the course direction, there was no significant decrease in the short-circuit current, whereas the open-circuit voltage exhibited a slight decrease. The output increased in a dry environment (20% relative humidity) and decreased in a humid environment (50% relative humidity). The adhesion stability of the coating was analyzed through the scanning electron microscopy (SEM) image of the Ni layer combined with the resistance-strain trend. They found that the coating was quite stable under a tensile strain of less than 50%, whereas damage appeared in the coating under a tensile strain of 100%. Based on this, the cycling performance of the TENG was tested under 50% strain. The result was promising, demonstrating that the current of the TENG textile did not decrease after 4000 cycles at 50% stretched strain. It is important to note that when the hydrophobic material was replaced by a hydrophilic material during the experiments, the device was damaged. Therefore, two factors limit the application of the TENG: strain and the material’s performance when wet.

The development of multilayer knitted fabrics is another attractive solution for manufacturing wearable flexible generators. Liu et al. [79] utilized a commercially available 3D-space fabric with a three-dimensionally penetrated structure that can offer spontaneous elastic space for pressing and releasing to fabricate a TENG. Thin PDMS film was carefully coated onto one surface of the 3D PET fabric directly. The CNT sheets were closely stacked onto the outer surface of the treated layer to serve as one electrode, whereas the other untreated surface was coated with a conductive silver paste to serve as the other electrode (Figure 6c). However, because the two layers were not friendly to human skin and the coating reduced the breathability, comfort, washability, stretchability, etc. of the fabric, the potential application of the device in daily wear is limited. During the experiment, the device was only made into insoles to collect people’s kinetic energy while walking.

Xiong et al. [80] proposed a synergetic triboelectric trapping layer of black phosphorus (BP) protected by a hydrophobic coating of cellulose oleoyl ester nanoparticles (HCOENPs) to alleviate degradation. By using these chemicals, they developed a durable skin-touch-triggered textile-TENG with a sandwiched structure. It was constructed using three fabric layers: a triboelectric fabric, a fabric electrode, and a waterproof fabric. By dip-coating or spray-coating, BP and HCOENPs were coated onto the PET fabric layer by layer in order to make the resulting triboelectric fabric, known as HCOENPs/BP/PET fabric (HBP-fabric). The silver flake mixed with PDMS was used as a as conducting medium and was coated onto the PET fabric via dip-coating to attain the fabric electrode. The waterproof fabric was created by dip-coating the HCOENPs onto a PET fabric. All three fabrics were stuck together with the aid of double-side tape, with the HBP-fabric in the middle. The all-fabric-based configuration delivered a conformable textile-TENG with extreme deformability, which worked well under 100% stretch conditions and had high durability. The output was maintained even after suffering 500 cycles of extreme deformations and 72 h of severe washing (Figure 6d). When mounted directly on skin or cloth, the TENG could fully fit different body regions, and it produced stable output voltages and current densities at different body locations.

The cover of the coating layer may make knitted devices lose their breathability and elasticity easily, and could even lead to skin-safety problems. The coating that Xiong et. al. developed solves the problems listed above, and even increases the aesthetic property of the knitted fabric device, making it possible to make patterned designs.

#### 2.2.3. 1D Devices Sewn into a Single Knitted Fabric

In addition to the above-mentioned wearable devices based on different fabric structures or textile technologies, there are some special generators that are single threads and were integrated into the fabric in the form of a stitch [81,82], cross stitch [83], or embroidery pattern [81]. As long as the mechanical properties of the yarn satisfy the sewing requirements, it can be fabricated into sophisticated textile structures and patterns. 

**Figure 7:**
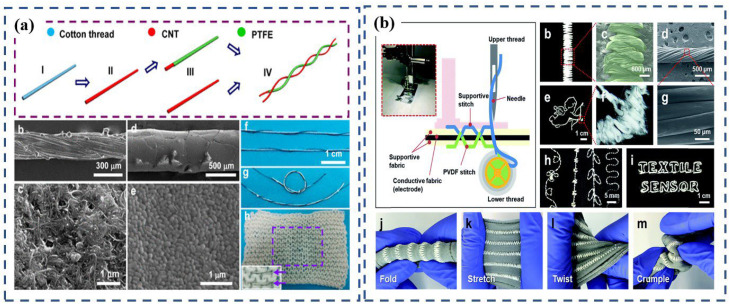
(**a**) Schematic diagram illustrating the fabricating process of an FBG that is made from CNT-coated cotton thread and PTFE thread. Reproduced with permission [84]. Copyright 2014, American Chemical Society. (**b**) PVDF stitch-based triboelectric textile sensors. Reproduced with permission [81]. Copyright 2018, The Royal Society of Chemistry.

In 2014, Zhong et al. [84] fabricated a metal-free fiber-based generator (FBG) using a cost-effective method that involved commercial cotton thread, a PTFE aqueous suspension, and carbon nanotubes as source materials (Figure 7a). This paper establishes the first proof of the idea that FBG can be sewn into knitted textiles (Figure 7a). FBG can extract energy from biomechanical motion to power a mobile medical system, making self-powered smart clothing possible. It can also convert biomechanical motion/vibration energy into electrical energy by using electrostatic effects, with an average output-power density of approximately 0.1 μW/cm^2^.

Shin et al. [81] reported using a sewing machine to stitch PVDF into programmable textile patterns for wearable self-powered triboelectric sensors. During their research, the PVDF thread was fabricated by dry-jet wet spinning and spin–draw. Then the PVDF thread was used as the lower thread and a commercial PET thread was used as the upper thread to stitch a design into knitted conductive fabrics, such as the simple stripe, embroidered pattern, embroidered lines, and even letters, indicating that PVDF threads are mechanically strong enough to be sewn into arbitrary patterns (Figure 7b). The stitch pattern is composed of twisted 5-ply PVDF threads. When stitched on stretchable fabric, the PVDF stitch patterns showed a good mechanical stability for folding, stretching, twisting, and crumpling deformations. The conductive fabric was sandwiched between supportive cotton fabrics, which were used as insulating layers. In order to demonstrate their applications in wearable devices, smart gloves and joint pads were manufactured based on PVDF-stitch triboelectric sensors. These wearable sensors can detect and distinguish different gestures and body actions by generating inherent signal patterns that represent specific gestures and actions. The device performance of the stitch sensor was retained without significant decrease even after repeated washing cycles, demonstrating that the proposed PVDF stitch sensor can be utilized for practical applications. In addition, compared to patterns that were coated on the fabric, the pattern in this wearable generator has better stability because it is stitched into the fabric. Moreover, combining this stitching technology with other wearable fabrics that can be tailored [26], manufactured on a large scale, and produced in large quantities [57] makes it possible to design garments that generate electricity.

The interlocking-loop structure of knitted fabric allows the coating material to cover the textile firmly and deeply. The existence of air gaps in knitted fabric can block the unlimited spread of a liquid coating material, meaning that it can be painted into various patterns as desired to meet the aesthetic requirements of various types of clothing. The addition of functional materials can directionally improve the relevant characteristics of the fabric and give new functions to the device. The patterned coating method instead of full coverage can not only retain the clothing characteristics of the original fabric, but also increase the aesthetic design. The knitted/coated-based generator shows a diversified development prospect.

### 2.3. Other Generators

**Figure 8:**
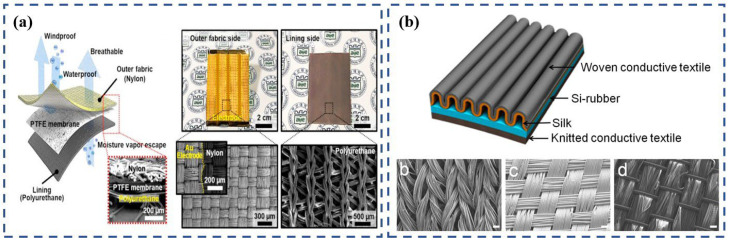
(**a**) Schematic of textile-TENG components (i.e., Gore-Tex); a woven-fabric composite consists of a nylon outer fabric, PTFE membrane (center layer), and polyurethane lining. Reproduced with permission [86]. Copyright 2014, Elsevier. (**b**) Corrugated textile-based triboelectric-structure generator consisting of a woven conductive textile, knitted conductive textile, and silk. Reproduced under the terms of the CC-BY Creative Commons [87].

Different fabric structures allow for different functions, so in some special and complex situations it is necessary to apply multiple fabrics at the same time [85]. Thus, hybrid textiles that are composed of a variety of structural fabrics have emerged. Each fabric in a hybrid textile performs its own duties to jointly create a resistance to the influences of a harsh environment. Inspired by this, Kim et al. [86] made a hybrid TENG that is based on woven fabric and knitted fabric (Figure 8a). The TENG is intended to be used as sportswear, so it has to make full use of the tightness and durability of woven fabrics and the soft and breathable properties of knitted fabrics. The basic structure of the waterproof and breathable textile-based TENG described in this study is composed of three distinctive layers. Using fabric woven with nylon as an outer layer grants both wind and water protection. A 50 μm-thick PTFE membrane (Gore-Tex) is the center layer, and the knitted polyurethane-based bottom lining with a breathable structure allows moisture to escape through the center-layer PTFE membrane. The flexible components of these TENGs were demonstrated easily when attached to clothes, effectively harvesting the motion energy of the human body.

Another study that analyzed the potential of a hybrid textile was that of Choi et al. [87], who proposed a corrugated textile-based triboelectric generator that can generate energy by stretching. The generator consists of woven conductive textile, silk, Si-rubber, and knitted conductive textile. The stretching process of the generator brings the silk and Si-rubber into full contact. The more friction and deformation that the silk and Si-rubber experience under high external mechanical force, the higher the quantity of triboelectric charges. This design cleverly puts the elastic characteristics of different fabric structures to use. Woven fabric is generally very poor in elasticity, so the woven components are made into a corrugated shape in this device to avoid breakage or fatigue and to increase the friction, resulting in a higher output. Knitted fabrics and silicone rubber are elastic and can be stretched from the original state to the fully stretched state, then spontaneously recover. This movement not only increases the friction, but also provides the driving force behind the device’s deformation (Figure 8b). Overall, this study provides a unique example of a hybrid woven-and-knitted generator that takes advantage of the elasticity of knitted fabric and bypasses the disadvantage of the inelasticity of woven fabrics. Moreover, the device can generate considerable energy from various deformations not only through pressing and rubbing but also by stretching. Additionally, the experiments demonstrated the generation of sufficient energy from various activities of a human body to power about 54 LEDs. These results demonstrate the potential application of the textile-based generator for self-powered systems. Table 1 provides a comparison of the electrical outputs of TENGs that were integrated into human clothing to collect human-motion energy in real-world circumstances.

The tightness, durability, and elasticity of knitted-structure fabrics differ greatly compared to those of woven-structure fabrics, which results in huge differences in water resistance, wind resistance, breathability, and stretchability of the two fabrics. The composite use of the two structures of fabrics can complement each other’s strengths and achieve a perfect synergy effect.

## 3. Summary and Outlook

Functional yarns with different properties are suitable for fabricating devices with different structures and designs. A woven structure has low requirements for the flexibility and length of the yarn. Woven fabrics have high durability and wear resistance, keep their shape well, and are often made into shirts, suits, denims, jackets, etc. Knitted structures have high requirements for the strength of the yarn because the yarn frequently suffers from the application of external force during the weaving process. Knitted fabrics have high elasticity and good air permeability, and are usually made into T-shirts, underwear, sportswear, socks, etc. Some special high-strength and high-flexibility functional yarns can not only be made into woven or knitted fabric devices but also into many more complex structures such as embroidery patterns, which greatly improves the ornamental potential and practicality of wearable devices.

For coated-fabric devices, coating on different fabric structures produces different fabric characteristics. The warp and weft yarns of the woven structure are usually arranged tightly, which gives the fabric a smooth surface that can easily be coated with functional materials. Unfortunately, the coverage of the coating generally results in a loss of air permeability for woven fabrics. As for knitted fabrics, the air gap between the loops provides enough space for the coating, allowing the coating to be applied to the yarn more deeply. The air permeability of coated/knitted fabrics will also decrease, but not as severely as for woven fabrics. Furthermore, most coatings are accompanied by drawbacks such as shedding, lack of breathability, and being unfriendly to the skin. The resulting issues of shortened life cycle, safety, and comfort need to be optimized by solving these problems, allowing yarn-based fabric generators to exhibit better mechanical strength and processability.

No matter what kind of structure the wearable device is, it must ultimately be integrated into clothing. Hence, the softness, breathability, comfort, washability, abrasion resistance, etc. of the device all need to be considered. Up to this point, the majority of research in this field has focused exclusively on the flexibility of the device, overlooking other practical properties. In recent years, more and more research projects have begun to test water washability, breathability, etc. with fabric pieces. So far, no one has been able to produce a garment where power-generating fabric forms the whole or main part of the clothing. The size, sweat safety, abrasion resistance, etc. are all obstacles to make power-generating garments. On one hand, increasing the output of the device is the main purpose of the structure design and material choice; on the other hand, the safety of the device is an issue that must be considered. For the human body, a safe voltage is below 36 V, a safe alternating current is 10 mA, and a safe direct current is 30 mA. If the voltage and current exceed the limit values at the same time, the device will be unsafe for human use. Although the power output produced by most devices up to this point has not been harmful to the human body, as further development pursues high-output devices, management and design used to control the limitation must be included in the device system.

Some efforts have been made in the field of device safety and practicality, such as the use of a waterproof coating layer [62,80,97,98], the application of a buck converter, and the integration of wearable supercapacitors [96,99], all of which are attempts to increase the safety and comfort of TENGs. In 2014, Zhao [100] set up a buck converter in the collection device to balance the gap between high voltage and low current, providing a possible reference for developing safety features in future electronic devices. The issues of humidity resistance [27], life cycle, and energy-conversion efficiency are also a concern. The research and development discussed above have increased TENGs’ resilience to wear, shear, water washing, extrusion, etc., but there is still area for improvement and increased testing.

Most of the research discussed in this paper focuses on integrating the generator into the upper garment; however, Proto’s [18] work showed that the kinetic energy produced by the lower body of humans is much higher than that of the upper body. Therefore, integrating generators into trousers to collect electricity is a possible direction for future development. Moreover, the energy collected from the human body is quite different from the energy collected in the laboratory, and increasing the comfort of a wearable generator is generally accompanied by a reduction in the amount of energy harvested [100]. A good balance between the comfort and actual output needs to be further explored. The current wearable devices under review have only been integrated into clothing in the form of small-area sheet fabrics, and the possibility of developing a fabric device that can be directly made into clothing should be investigated further. Smart power-generating clothing gives a new meaning to our clothing and provides a green and renewable way to collect electricity.

## Figures and Tables

**Table 1:** List of the reported textile-based TENGs with different performance.

Ref	Location of Energy Collection	Current	Voltage	Charge Accumulation	Materials	Device Substrate	Combination with Garment
[6]		19 μA	900 V	203 mWm^−2^	PTFE	Polyester fabric	Knitted
[7]	Under foot, under arm, elbow joint	4 μA	50 V	3.7 μC min^−1^	Ni-cloth, parlyene	Polyester fabric	Attached
[26]	Underneath the arm	1.4 μA	113.21 V	80 mW/m^2^	PTFE, PVDF	Nylon, silk, cotton, T/C, PET, PP fabric	
[54]	Underneath the arm	0.91 mA	12.6 V	11.92 mA cm^−2^	EVA, PDMS	EVA tubes	Attached, woven
[58]	Underneath the arm	1.5 μA	~118 V	48 nC	Conductive carbon wires	Carbon and PTFE textile	
[63]	Underneath the arm	40 μA			Ni and parlyene	Polyester yarns	Woven
[66]	Elbow	12 μA	500 V	310 μW/cm^2^	Silk fibroin, PVDF	Conductive fabrics	
[67]	Sleeve, underneath the arm	55 μA	100 V		Ni and parlyene	Polyester fabric	Attached
[75]		2.9 μA	150 V	85 mW·m^−2^	Stainless steel, polyester	Silicone, rubber	Knitting
[77]	Chest	1.8 μA	49 V	50.6 mF cm^−2^, 94.5 mW m^−2^	Ni, rGO-Ni	Stretchable polyester fabric	Sewn
[88]	Hand	0.25 mA	80 V		PTFE and copper, ZnO and copper	Polymer textile	
[89]	Between forearm, human body	0.2 mA	2 kV	69 μC/s	Nylon, Dacron	Cotton	
[90]	Under arm	0.4 μA	40 V	0.18 μW/cm^2^, 85.2 mF/cm^2^	PI, PU, Al, PDMS, CNT/RuO_2_ PVA/H_3_PO_4_	Carbon fabric	
[91]	Pocket, sleeve	65 μA	120 V		ZnO, PDMS	Ag-coated knitted textile	Attached
[92]	Elbow, knee		4.16 V		Metal, conductive fiber, AgNWs	Nylon fiber, silicone, rubber tube	
[93]	Chest, hand, wrist				CNT	Polyester, nylon textile	Sewn
[86]		4 μA	120 V	68 μW m^−2^	Au nanodots, polyurethane/PTFE	Nylon woven-fabric	
[94]		399.42 mA	17 V		Copper	Polyester filament	Sewn
[81]	Wrist, elbow, ankle, knee	190 nA	1.8 V		PVDF, Al	Nylon fabric	Sewn, stitched
[95]	Butt, underneath the arm, arm, knee		540 V	2 Wm^−2^	PEDOT: PSS, PTFE or silicon rubber	Cotton textile	Attached
[96]	Sleeve	3 μA	60 V	≈78.1 μWh cm^−2^, 14 mW cm^−2^	Ni/Cu, rGO/CNT, NiCo BOH	Polyester yarn	Woven, knitted

## Data Availability

No new data were created or analyzed in this study. Data sharing is not applicable to this article.
